# Anatomical Variations of Superior Sagittal Sinus and Tributary Bridging Veins: A Cadaveric Study

**DOI:** 10.7759/cureus.21979

**Published:** 2022-02-07

**Authors:** Raimondas Juskys, Saulius Rocka, Andrej Suchomlinov

**Affiliations:** 1 Faculty of Medicine, Vilnius University, Vilnius, LTU; 2 Department of Anatomy, Histology, and Anthropology, Faculty of Medicine, Vilnius University, Vilnius, LTU

**Keywords:** brain anatomy, parasagittal region, cadaveric dissection, bridging veins, superior sagittal sinus

## Abstract

Background and objective

Injuries to the parasagittal cerebrovenous structures may lead to devastating complications. Being aware of the inherent anatomical heterogeneity in the region might lower the rate of undesirable outcomes. In this study, our goal was to characterize the superior sagittal sinus (SSS) positioning in relation to the midline and depict tributary bridging veins (BVs) distribution over the lateral surface of the cerebral hemispheres.

Methods

We performed anatomical dissections of the brain in 10 cadaveric specimens (five females and five males; median age: 52 years, range: 44-74 years). Measurements (in mm) of the SSS width and deviation of its lateral margin from the midline were obtained along the entire length of the structure at six craniometric points [at mid-distance between Nasion and Bregma (½ N-B); at Bregma (B); in the middle of the Bregma-Lambda segment (½ B-L); at Lambda (L); halfway between Lambda and Inion (½ L-I); and at Inion (I)]. The count, diameter, and lateral insertion points of the draining BVs were also documented in three segments [Nasion-Bregma (N-B), Bregma-Lambda (B-L), and Lambda-Inion (L-I)].

Results

The width of the SSS increased progressively along the direction of the blood flow (p<0.01). There was an SSS lateral deviation bias to the right, but the comparison failed to reach the significance level (p=0.12). The maximal lateralization of the SSS in the pre-Lambdal interval was 13.1 mm on the right side and 11.7 mm on the left side. These values increased up to 19.8 mm and 15.1 mm in the torcular area on the right and left sides, respectively. A total of 191 BVs were identified (a mean of 19.1 ± 2.5 per individual). The L-I segment showed a lower number of BVs as compared to its N-B and B-L counterparts (mean: 0.9 ± 0.6 vs. 8 ± 1.8 and 10.2 ± 2, respectively, p<0.01). Along the entire span of the SSS, the average diameter of the BVs was larger on the right side (mean: 1.4 ± 0.9 mm vs. 1.1 ± 0.8 mm on the left, p<0.01). The average lateralization of BVs dural entry points was lower on the left side in the B-L segment (mean: 5.6 ± 6.4 mm vs. 8.8 ± 6.7 mm on the right, p<0.01). There was a statistically significant trend of decreasing BVs lateralization with each consecutive SSS segment (mean: 10.9 ± 7.4 mm in the N-B segment, 7.3 ± 6.7 mm in B-L, and 1.6 ± 1.2 mm in L-I, p<0.01). The maximal lateral deviation of BVs insertion points was 33.6 mm in N-B, 30 mm in B-L, and 4.1 mm in L-I portions of the SSS.

Conclusions

In most cases, the SSS deviated laterally from the midline, up to 13 mm in the pre-Lambdal segment and up to 20 mm in the torcular area. Right-sided BVs were of larger average diameters. The lateral insertion points of BVs decreased along the rostrocaudal span of the SSS.

## Introduction

A good understanding of the neurosurgical anatomy is a crucial prerequisite for optimizing outcomes and limiting the frequency of intra- and postoperative complications. The superior sagittal sinus (SSS) is a caudally-expanding dural structure collecting venous blood from the medial parts of the fronto-parieto-occipital cortex and the basal surface of the frontal lobe. Generally, superficial cortical veins, also known as bridging veins (BVs), are responsible for the vast majority of the volumetric contribution to the SSS [1].

Although permanent neurological sequelae after venous drainage obliteration are less common in comparison to the arterial system, (in)advert interruption of cortical venous outflow nonetheless might lead to potentially preventable complications such as bleeding, cerebral edema, infarction, and ultimately severe neurological outcomes. Similarly, besides the hazards associated with the BVs termination, injury to the SSS might expose the patient to the risk of an air embolism, requiring a technically-demanding surgical repair, and, if unsuccessful, ligation of the SSS in order to prevent detrimental hemorrhage [2,3]. The risk of profuse bleeding and permanent neurological deficits increases exponentially as more tributaries pool into the dural structure [4]. Therefore, substantial knowledge of anatomical peculiarities in the region is an important requirement for every neurosurgeon operating on the parasagittal lesions.

The aim of this study was to delineate variations in the SSS and BVs anatomy in a formalin-fixed cadaveric cohort. Particular attention was directed towards the SSS positioning in relation to the midline and detailed topographical mapping of the BVs. Our findings could be of interest to neurological surgeons, neuroanatomists, and researchers engaged in the field.

## Materials and methods

A total of 10 formalin-fixed adult cadavers of Lithuanian descent (five males and five females) from the Anatomy, Histology, and Anthropology Department of the Vilnius University, Lithuania were enrolled in the study. All participants had signed the informed consent prior to the death. None of the individuals showed any evidence of disrupted craniocerebral anatomical continuity (e.g., previous craniotomy, skull fractures, craniosynostosis, or cerebrovenous pathology). Following the soft tissue dissection of the scalp, a midline was identified, representing a line connecting Nasion and Inion in the mid-longitudinal plane. The length of three segments was documented prior to bony removal: the distances between Nasion-Bregma, Bregma-Lambda, and Lambda-Inion (N-B, B-L, and L-I segments, respectively). Finally, intracranial structures were exposed by performing a bilateral craniectomy with the preservation of sagittal, coronal, and lambdoid sutures. All of the subsequently mentioned measurements were performed three times by the same author (R.J.) by using a standardized digital caliper with a <0.01-mm accuracy.

**Figure 1:**
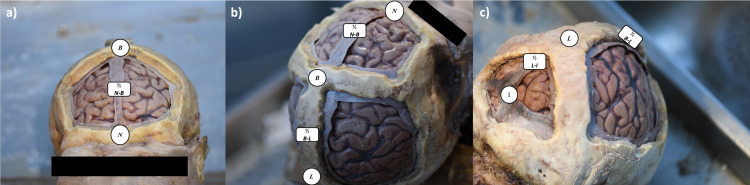
Cadaveric specimen after bony and dural removal Along the length of superior sagittal sinus, six craniometric points were identified: Nasion (N), mid-distance between Nasion and Bregma (½ N-B), Bregma (B), in the middle between Bregma and Lambda (½ B-L), Lambda (L), halfway between Lambda and Inion (½ L-I), and Inion (I)

The SSS was measured at six craniometric points in terms of 1) width and 2) lateralization (defined as the distance from the midline to the lateral edge of the SSS). The landmarks at which measurements were performed were as follows: 1) at mid-distance between Nasion and Bregma (½ N-B); 2) at Bregma (B); 3) in the middle of the Bregma-Lambda segment (½ B-L); 4) at Lambda (L); 5) halfway between Lambda and Inion (½ L-I); and 6) at Inion (I). Figure 1 presents additional information about the process. Negative and positive lateralization values were used to describe leftward and rightward deviations of the SSS, respectively. Additionally, the side of the dominant transverse sinus was also documented.

**Figure 2:**
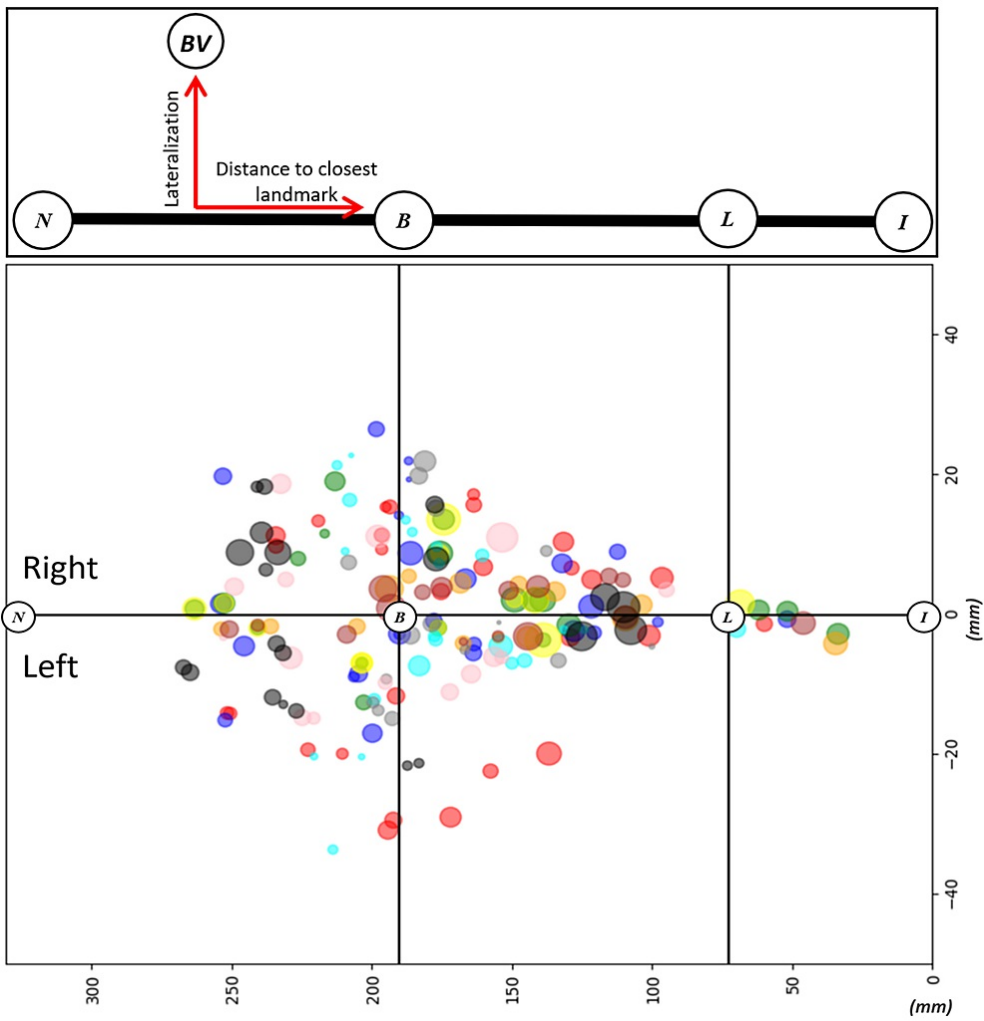
Schematic representation of the methodology used for measurements acquisition (top part) and map of the bridging veins (bottom part) Each color represents an individual cadaver data. The diameters of the circles are directly proportional to the caliber of the cortical vein. The length of each segment (N-B, B-L, and L-I) is visualized proportionally to the calculated average in our sample. Distances on the X-Y axis are depicted in millimeters (mm) N: Nasion; B: Bregma; L: Lambda; I: Inion

The evaluation of BVs included their 1) count, 2) diameter, and 3) lateralization (defined as the distance from the midline to the point where the tributary pierces the dura mater). The same aforementioned segments (N-B, B-L, and L-I) were used for the group analysis. Additionally, the distance to the closest craniometric landmark (B or L) was documented for each specimen in order to obtain the positional data of each tributary on recreated two-dimensional X-Y axis map of the BVs. Measurements of lateralization and distance to the closest craniometric landmark were used for the function (see Figure 2 for additional information).

The data were analyzed using the SAS/STAT software (SAS Institute, Cary, NC) by employing descriptive and hypothesis-testing statistical methods. Normality of the data was defined using the Kolmogorov-Smirnov test, whereas variance was assessed with Bartlett’s test. Two-sample Student’s t-test was used to compare normally distributed and equal variance variable means between two groups of interest. Similarly, the Mann-Whitney U test was employed for skewed data. For comparison of more than two independent groups, ANOVA (for normally distributed data) and Kruskal-Wallis ANOVA (for unequally distributed data) tests were utilized. Central tendency measures are reported as mean ± SD or median ± IQR for parametric and non-parametric counterparts, respectively. The null hypothesis was rejected if the p-value was <0.05 in two-sided analysis. Final results are reported with one digit after the decimal point. All measurements were made and are reported in mm units.

## Results

The median age of our sample was 52 years (range: 44-74 years), and there were no statistically significant differences in measurement results between the sexes. Apart from one individual with a leftwardly placed sagittal suture, there were no other abnormalities in sutural anatomy. The median length of the N-B segment was 132.7 ± 2.4 mm, the B-L distance was 123.1 ± 10.3 mm, and the L-I interval equated to 82.3 ± 8.2 mm.

The results of SSS measurements are summarized in Table 1. The width of the SSS increased in the rostrocaudal direction (p<0.01). A similar tendency was observed in terms of SSS rightwards divergence from the midline while extending dorsally. Anyway, there was no statistically significant difference in the lateralization of the SSS while comparing the difference in medians between each craniometric measurement point result (p=0.12). Although the smallest deviation was observed at the ½ B-L measurement point (median 4.4 ± 13.1 mm), this area was characterized by the highest variability (widest IQR of ± 13.1 mm) in the results obtained. The maximal lateralization of the SSS to the right ranged from 10.3 to 13.1 mm up to the L craniometric point but extended as much as 17.2 to 19.8 mm in the L-I segment. Excluding the torcular area, the maximal lateral deviation of the SSS to the left was 11.7 mm at the L landmark. This value increased up to 15.1 mm at the confluence of the sinuses area. In 8/10 specimens, the dominant transverse sinus was found on the right side.

**Table 1:** Summary of the superior sagittal sinus (SSS) measurements at six craniometric points *Negative values in the reported range represent a maximal leftward displacement of lateral margin, while positive values reflect maximal rightward deviation. N: Nasion; B: Bregma; L: Lambda; I: Inion; ½ N-B: midway between N and B; ½ B-L: midway between B and L; ½ L-I: midway between L and I

Measurement	½ N-B	B	½ B-L	L	½ L-I	I
Width in mm, median ± IQR (range)	7.3 ± 3.3 (4.4–12.9)	10.2 ± 2.5 (8.2–13.1)	11.9 ± 1.9 (6.7–15.4)	13 ± 2.3 (9–16.4)	11.9 ± 4.2 (9–19.8)	15.6 ± 4.7 (11.2–23)
Lateralization in mm, median ± IQR (range)*	5.5 ± 2 (-2.7–10.4)	7.3 ± 4 (-9.4–10.3)	4.4 ± 13.1 (-9.9–10.7)	9.7 ± 3.2 (-11.7–13.1)	9.8 ± 12 (-11–17.2)	12.4 ± 5.6 (-15.1–19.8)

The results of BVs measurements are summarized in Table 2. A net total of 191 veins were found in our sample bilaterally (on average 19.1 ± 2.5 per individual, 51.8% of which were found on the left side). Percentage-wise, the N-B segment accounted for 41.9%, B-L for 53.3%, and L-I for 4.7% of all tributaries draining to the SSS. There was no statistically significant difference in BVs count while comparing hemispheric sides per each segment. Comparison of different segments revealed a meaningfully lower number of BVs in the L-I segment as compared to N-B and B-L segments (on average, 0.9 ± 0.6 vs. 8 ± 1.8 and 10.2 ± 2, respectively, p<0.01). Along the entire length of the SSS (in the N-I segment), the average caliber of the tributaries was larger on the right side (mean diameter of 1.1 ± 0.8 mm on the left vs. 1.4 ± 0.9 mm on the right, p<0.01). Anyway, in the subgroup analysis of each segment, this relationship remained evident only in the N-B segment (mean diameter on the left 0.8 ± 0.4 mm vs. 1.2 ± 0.8 mm on the right, p<0.01). Finally, there was a statistically significant difference in the lateralization of BVs dural entry points on the right side of the brain in the B-L segment (mean lateral deviation on the left was 5.6 ± 6.4 mm vs. 8.8 ± 6.7 mm on the right, p<0.01). There were no such relationships observed between left and right sides in other segments. A comparison between each of the three segments showed a strong, statistically significant trend of decreasing BVs lateralization with each consecutive segment (mean lateral deviation in the N-B segment was 10.9 ± 7.4 mm, in the B-L segment: 7.3 ± 6.7 mm, and in the L-I portion: 1.6 ± 1.2 mm, p<0.01). The maximal lateral deviation of the tributaries was observed in the N-B segment as well, extending up to 33.6 mm from the midline. Similarly, the highest lateralization in the B-L portion of the SSS was 30 mm, whereas the L-I segment showed only 4.1 mm as the highest value.

**Table 2:** Summary of bridging veins measurements Statistically significant differences (p<0.05) between variables in two or more groups are bolded and highlighted by superscripted numbers ^1, 2^Statistically significant difference in the total count of BVs when comparing the L-I segment with N-B^1^/B-L^2^ segments, respectively (p<0.01 in both comparisons) ^3, 4^Statistically significant difference in the mean diameter of BVs between hemispheric sides in the N-B segment^3^ and along the entire length of SSS^4^ (N-I segment, p<0.01 in both comparisons) ^5^Statistically significant trend showing decreasing average distance from the midline to the lateral insertion points of BVs with each consecutive SSS segment (N-B, B-L, and L-I, p<0.01) ^6^Statistically significant difference in the lateralization of BVs drainage points between hemispheric sides in B-L segment (p<0.01) BV: bridging vein; N: Nasion; B: Bregma; L: Lambda; I: Inion

BVs measurement	N-B segment			B-L segment			L-I segment			N-I segment		
	Left	Right	Total	Left	Right	Total	Left	Right	Total	Left	Right	Total
Count, mean ± SD (range)	4.5 ± 1.5 (3–7)	3.5 ± 2 (1–7)	8 ± 1.8 (4–14)^1^	4.8 ± 2 (2–8)	5.4 ± 2 (2–9)	10.2 ± 2 (6–17)^2^	0.6 ± 0.5 (0–1)	0.3 ± 0.7 (0–2)	0.9 ± 0.6 (0–3)^1,2^	9.9 ± 2.4 (5–14)	9.2 ± 2.7 (6–15)	19.1 ± 2.5 (12–28)
Diameter in mm, mean ± SD (range)	0.8 ± 0.4 (0.1–1.9)^3^	1.2 ± 0.8 (0.1–3)^3^	1 ± 0.6 (0.1–3)	1.2 ± 1 (0.1–4.6)	1.4 ± 0.9 (0.1–4)	1.3 ± 0.9 (0.1–4.6)	1.5 ± 0.5 (0.9–2)	2 ± 0.7 (1.6–2.9)	1.7 ± 0.6 (0.9–2.9)	1.1 ± 0.8 (0.1–4.6)^4^	1.4 ± 0.9 (0.1–​​​​4)^4^	1.2 ± 0.8 (0.1–4.6)
Lateralization in mm, mean ± SD (range)	11 ± 7.8 (1.5–33.6)	10.7 ± 7 (0.9–26.6)	10.9 ± 7.4 (0.9–33.6)^5^	5.6 ± 6.4 (0.3–28.9)^6^	8.8 ± 6.7 (1.1–30)^6^	7.3 ± 6.7 (0.3–30)^5^	2 ± 1.3 (0.6–4.1)	1 ± 0.6 (0.5–1.7)	1.6 ± 1.2 (0.5–4.1)^5^	7.8 ± 7.4 (0.3–33.6)	9.3 ± 6.9 (0.5–30)	8.5 ± 7.2 (0.3–33.6)

Figure 2 presents all BVs identified in our sample. Each circle refers to the individual BV; a diameter is proportional to the caliber of the vessel, and color-coding is used to separate each cadaver’s measurement results. The central horizontal line represents the midline, whereas vertical intersecting lines mark each craniometric landmark (B and L respectively). Both lateral deviation and distance to the closest craniometric point were used to generate a venous tributary map.

## Discussion

A good understanding of the possible cerebrovenous anatomical variations in the parasagittal region plays an important role in surgical planning and execution [1]. To our knowledge, the earliest attempt to systematically describe the SSS and tributary superficial veins was made in 1934 by O'Connell [5]. Although many additional cadaveric studies have been published since then, probably the most comprehensive manuscript on the topic was drafted by Rhoton and colleagues in 2002 [1,6-10]. In addition, recent technological advances in neuroimaging have enabled the further assessment of the anatomical peculiarities in a non-invasive manner, albeit the topic of direct comparison of the results between the two methodological approaches could be brought up for a debate [11-13].

Essentially, our study reiterated a previously described understanding regarding the tendency of the SSS to increase in size and deviate laterally as the structure extends dorsally [8-10,13]. Obviously, an enlarging lumen of the SSS allows for the accommodation of an increasing volume of venous blood draining from the cerebral tissue along the length of the dural venous system. In the majority of the studies reported, the SSS width seemed to be largest at the L craniometric point, ranging from 8 to 12 mm in different publications [8-10,13]. These findings are in line with our observations of the median SSS width of 13 mm at the L measurement point. Anyway, the widest SSS area in our sample was in the torcular region, with a median SSS horizontal diameter of 15.6 mm.

Only two other studies have also addressed the SSS width beneath the external occipital protuberance, respectively reporting an average of 7.8 mm and 11.6 mm in SSS diameters [10,13]. An interesting inverse relationship was reported by Sayhan et al., as they found a decreasing SSS width as measured at B, vertex, and L craniometric points [9]. Another important point to be stressed is that instead of the sagittal suture, we decided to use the midline for SSS lateral deviation referencing. Such a methodology was favored given the ease of its identification in surgical practice, applicability along the whole SSS length (as opposed to the B-L segment only), and the possibility of morphological alterations and/or paramedian positioning of the sagittal suture [14,15]. We managed to identify only one other study that used the midline as a landmark for the localization of SSS [13]. Reis et al. used five craniometric points (similar to our six landmarks, except for the ½ L-I area) at which the lateralization of the SSS was classified as either in the midline, displaced to the left, or displaced to the right in the reference to the midline. In all the eight cadaveric specimens used in the study, the SSS diverged from the longitudinal plane consistently, and this trend accelerated along the direction of the blood flow. The right-sided deviation was the predominant finding. Our data support these conclusions, as similar results were found in this cadaveric cohort. In addition, the authors underlined that the SSS could deviate up to 10 mm sideways from the midline, although the exact ranges in measurements were not reported [13]. Based on our study, the SSS lateral margin extended from 12 mm on the left to 13 mm on the right in the pre-L portion of the SSS. These values increased up to 15 mm on the left and up to 20 mm on the right hemispheres in the post-L segment of the SSS, emphasizing the need for extra diligence during surgical manipulations in the posterior parietal and occipital parasagittal areas. Moreover, the lateral edge of the SSS at the ½ B-L craniometric point was characterized by the largest spread around the mean, reflecting higher heterogeneity in the measurement results and, therefore, potentially less consistent anatomical perpetuity.

The second part of our study focused on superficial cortical veins draining to the SSS. Primarily, there is a significantly lower number of veins emptying to the L-I portion of the SSS, even after adjusting for the shorter length of the segment. These findings are consistent with other similar studies [8-12]. Furthermore, an interesting observation was that in addition to the post-L section, we consistently noticed that there was an area ~1 cm proximal to the B that is also relatively devoid of the BVs (see Figure 2 for the illustration). The latter finding was explicitly described only in one other study, where the authors defined the region as an anterior nontributary segment of the SSS, measuring on average ± 0.7 mm of the Bregmal vicinity [11].

In terms of the BVs diameter along the whole SSS length, right-sided tributaries were almost 30% larger as compared to the left-sided equivalents. Subsegmental analysis revealed the same relationship in the N-B portion, and there was a similar tendency in the B-L interval, albeit the latter failed to reach statistical significance (p=0.15). Unfortunately, we did not have any additional information regarding the hemispheric dominance pre-mortem, and hence a potential link between venous drainage patterns and functional lateralization remains to be determined. None of the previous studies on the topic have reported disparities in side predilection [9,11]. The largest number of and greatest diameter veins were mostly concentrated in the Rolandic region, likely reflecting the relatively higher metabolic demand of the area. Frequently, we observed a dominant vein of Trolard draining the paracentral neural tissue, with the largest measured diameters of 4-4.6 mm on the left and right sides, respectively. The phenomenon of venous clustering around the central sulcus was also previously described by other authors [8,9,11].

Finally, we addressed the question of BVs lateralization in terms of dural entrance over the convexity. There was a clear tendency for the tributaries to empty more medially along the rostrocaudal direction, with a statistically significant difference between each segment (p<0.01). In addition, the B-L segment analysis revealed a larger tributary lateralization propensity over the right hemisphere (p<0.01). Generally, we found that the majority of the vessels joined the dural route up to 20 mm for the N-B segment, 15 mm for B-L, and 4 mm for L-I, lateral to the midline. Anyway, several outliers were identified, extending from the midline as far as 33.6 mm and 30 mm in N-B and B-L segments, respectively. Of note, far laterally inserting cortical veins either joined meningeal sinuses or adhered to the inner surface of dura mater before coursing medially towards the SSS. We did not notice a direct tributary communication with the venous lacunae, albeit this statement remains anecdotal as the issue was out of the scope of our investigation. Besides the empirical observations portrayed by Rhoton’s group, where BVs were found to extend up to 30 mm laterally, we failed to identify any studies systematically addressing the question of tributary lateralization [1].

It is important to emphasize that there is a considerable difference in the methodologies between the different studies we discussed. In the present study, we examined formalin-fixed cadavers, and our measurement results could diverge from other studies that employed fresh or silicon-injected cadaveric material. Similarly, technical diversity and high inter-observer variability are common issues limiting the comparison of the results.

Limitations

The generalizability of our findings is limited by the small sample size and possible ethnical considerations. In addition, we did not approach the topic from all possible angles, as meningeal sinuses and venous lacunae morphological variations and/or related BVs variables (such as angulations or emptying localizations) also play an important role in the all-around characterization of the parasagittal anatomy. These structures could form a compelling basis for further anatomical studies in the region.

## Conclusions

The localization of the SSS in relation to the midline and distribution of BVs varies significantly between individuals. In the majority of cases, there is a tendency for the SSS to deviate laterally along the direction of the blood flow, usually to the right. In our sample, the SSS lateral margin extended up to 13 mm in the pre-L segment and up to 20 mm in the confluence area. The right hemispheric side harbored BVs of larger diameter. Lateral insertion points of superficial cortical veins decreased with each consecutive segment, with a maximal deviation of up to 33.6 mm, 30 mm, and 4.1 mm in N-B, B-L, and L-I portions of the SSS, respectively.
